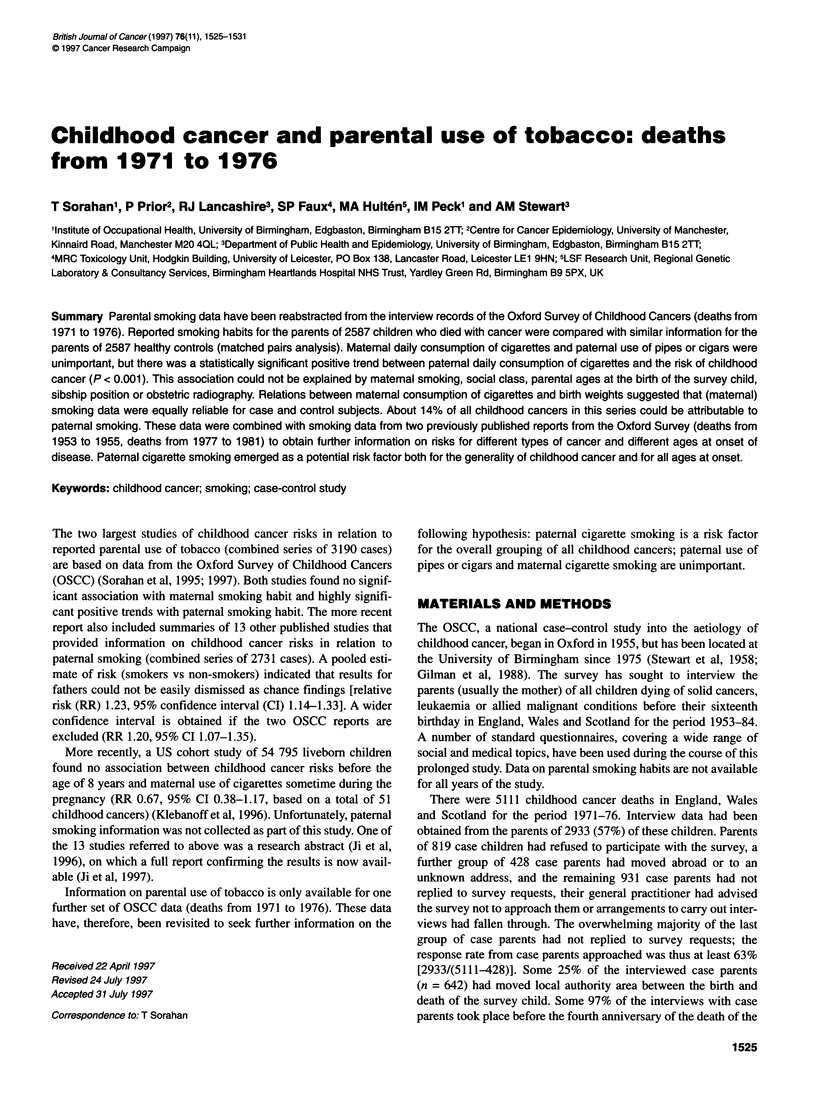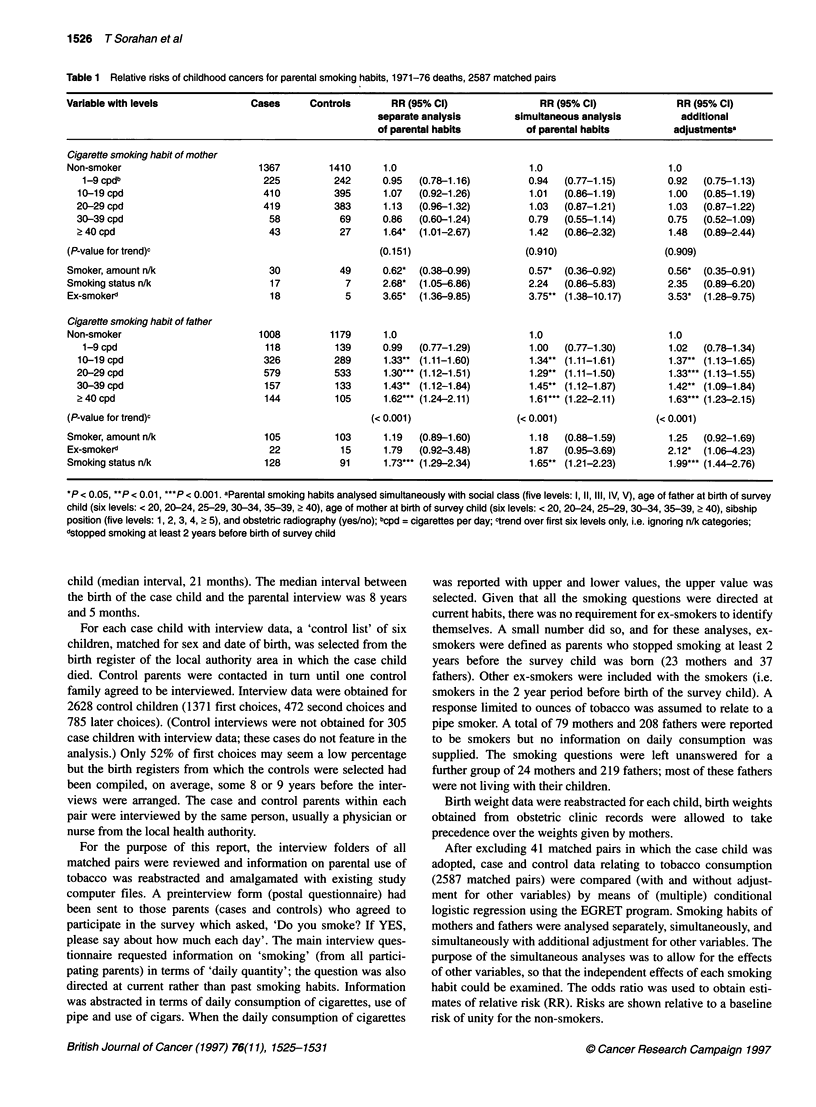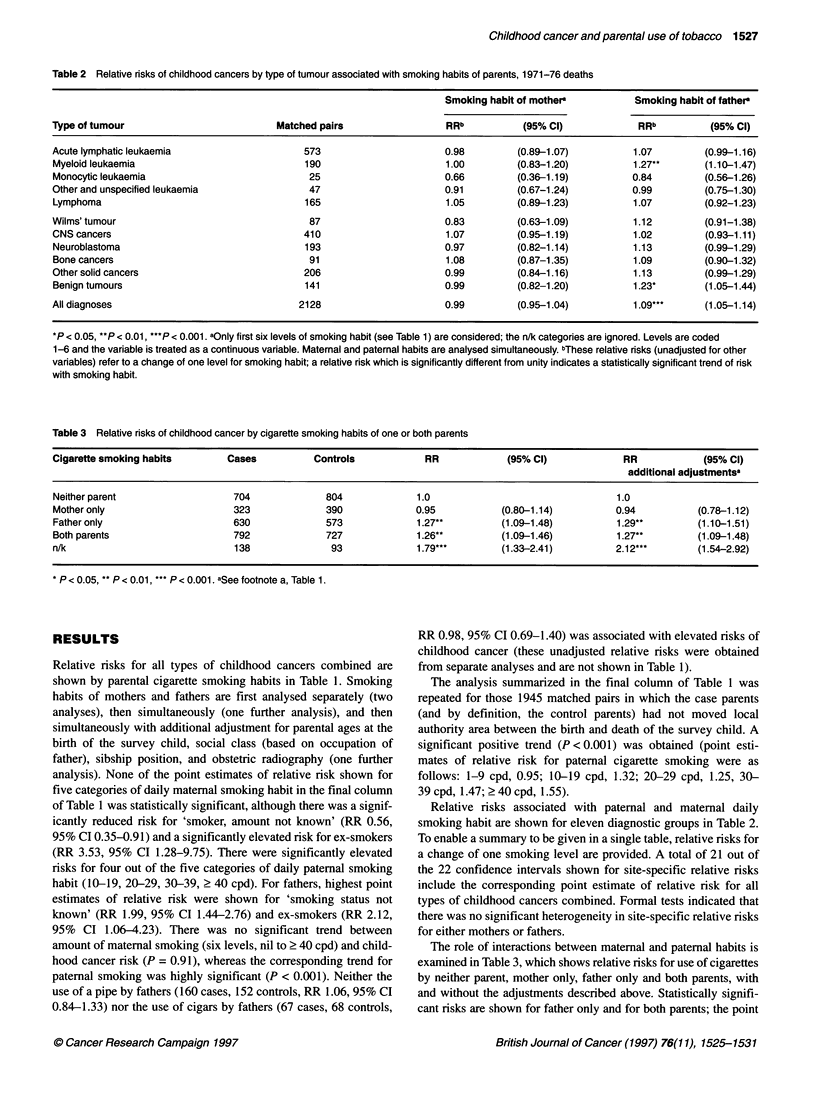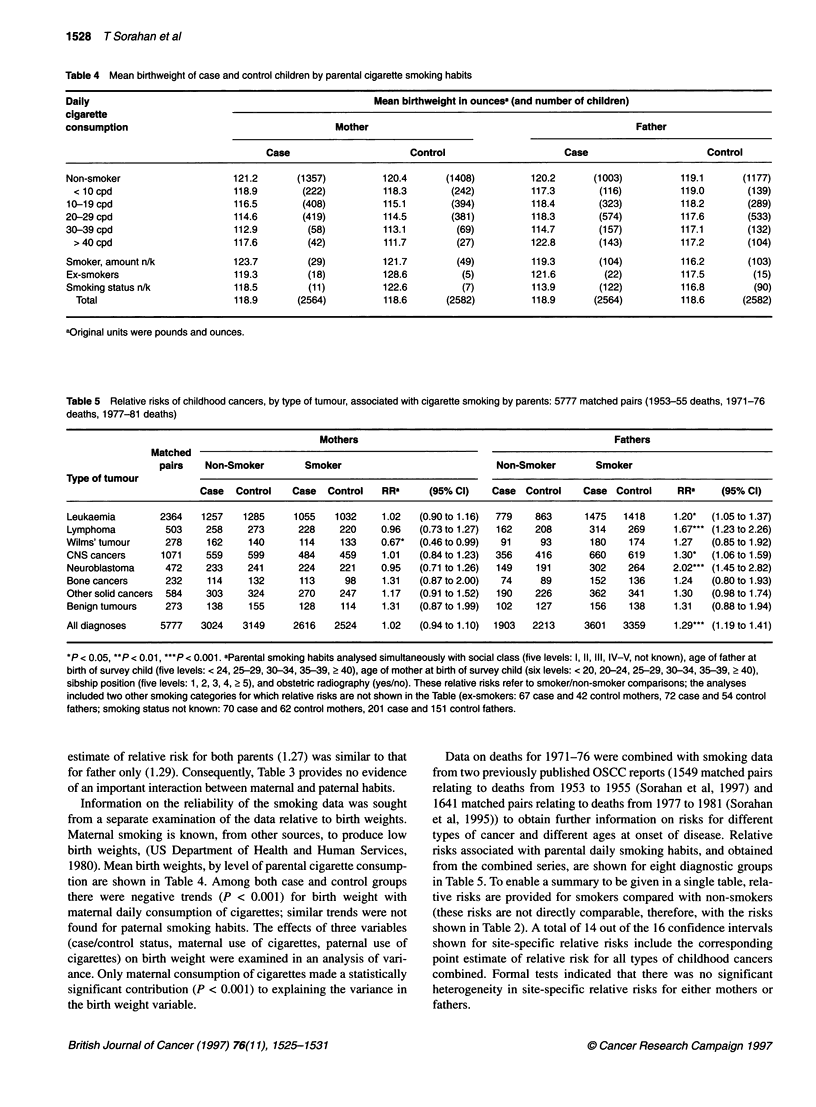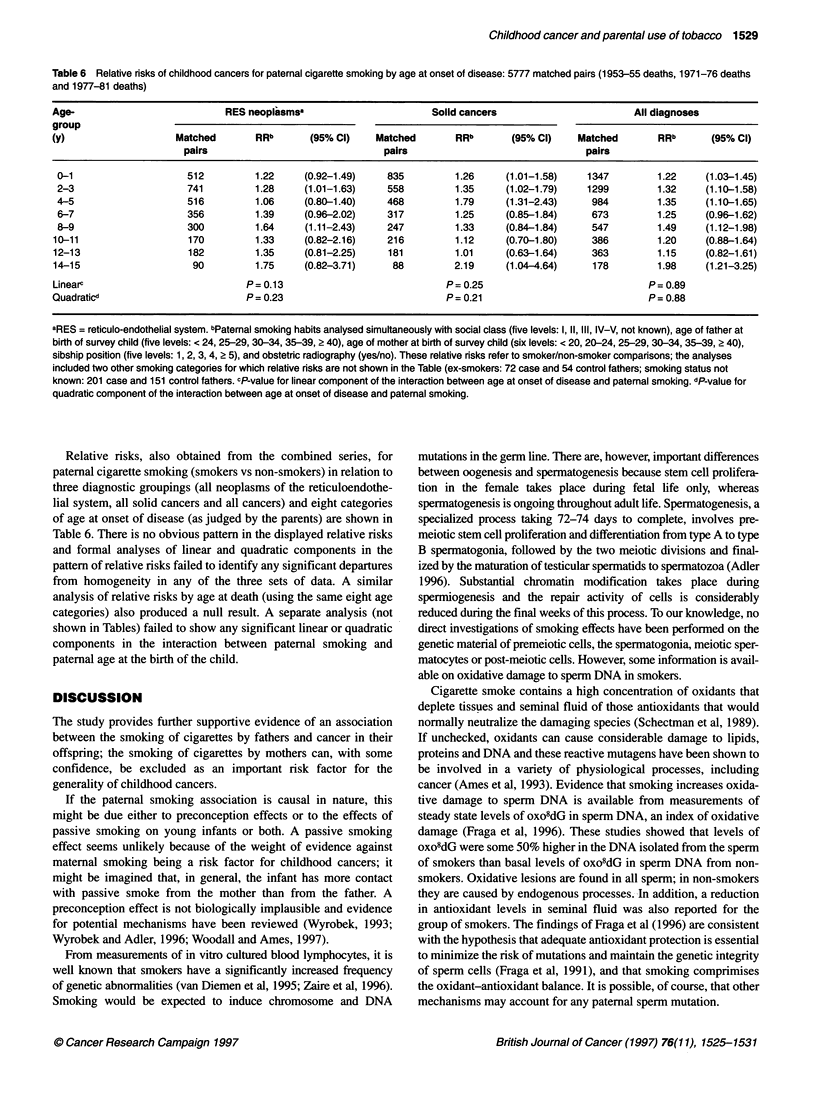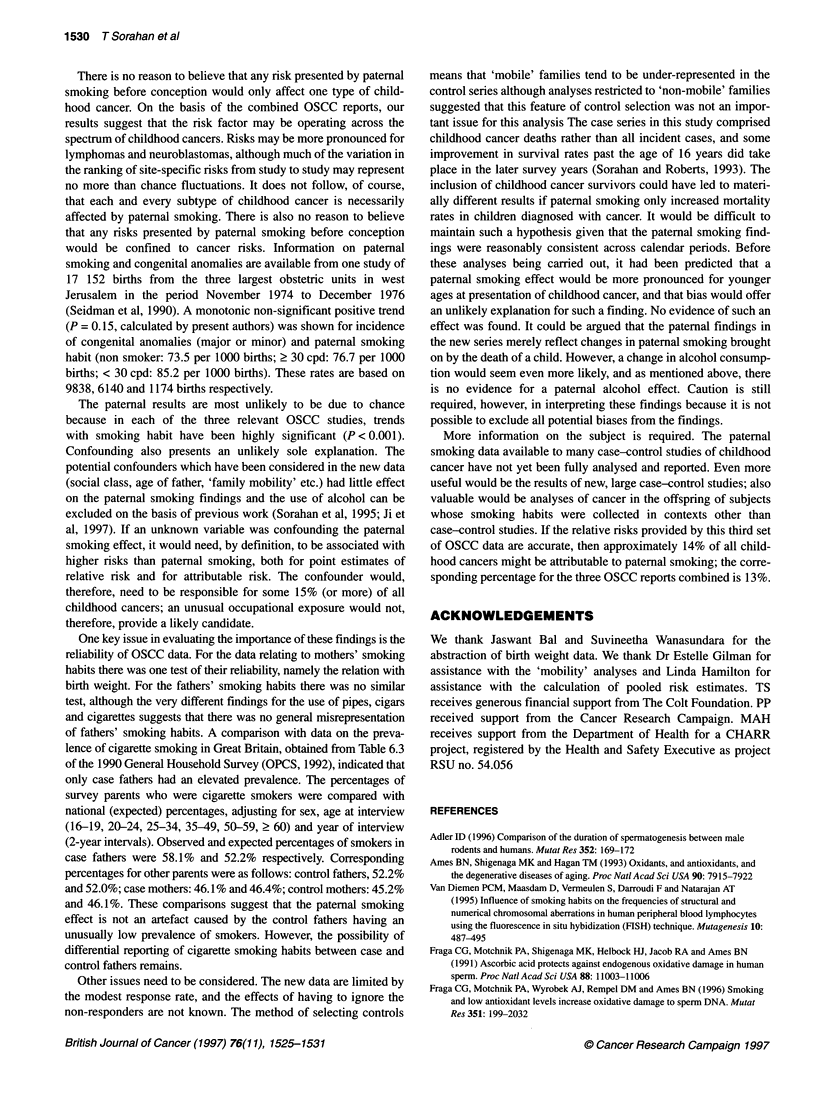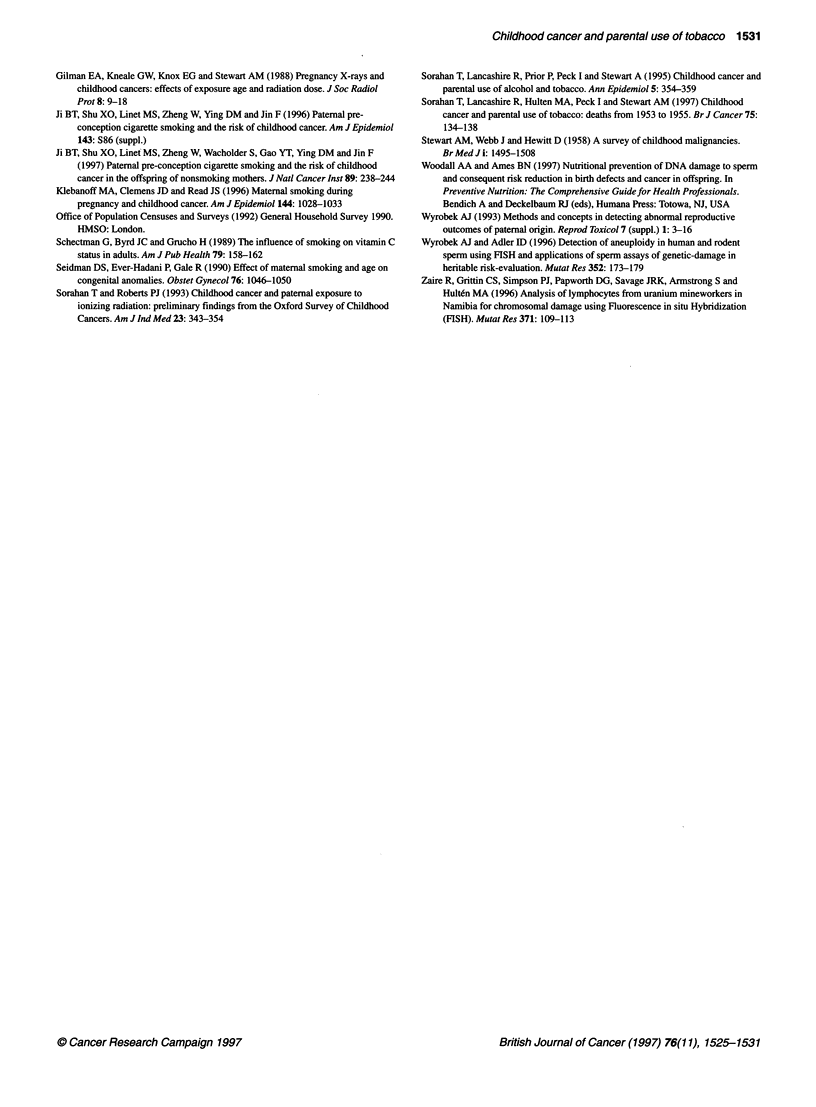# Childhood cancer and parental use of tobacco: deaths from 1971 to 1976.

**DOI:** 10.1038/bjc.1997.589

**Published:** 1997

**Authors:** T. Sorahan, P. Prior, R. J. Lancashire, S. P. Faux, M. A. HultÃ©n, I. M. Peck, A. M. Stewart

**Affiliations:** Institute of Occupational Health, University of Birmingham, Edgbaston, UK.

## Abstract

Parental smoking data have been reabstracted from the interview records of the Oxford Survey of Childhood Cancers (deaths from 1971 to 1976). Reported smoking habits for the parents of 2587 children who died with cancer were compared with similar information for the parents of 2587 healthy controls (matched pairs analysis). Maternal daily consumption of cigarettes and paternal use of pipes or cigars were unimportant, but there was a statistically significant positive trend between paternal daily consumption of cigarettes and the risk of childhood cancer (P < 0.001). This association could not be explained by maternal smoking, social class, parental ages at the birth of the survey child, sibship position or obstetric radiography. Relations between maternal consumption of cigarettes and birth weights suggested that (maternal) smoking data were equally reliable for case and control subjects. About 14% of all childhood cancers in this series could be attributable to paternal smoking. These data were combined with smoking data from two previously published reports from the Oxford Survey (deaths from 1953 to 1955, deaths from 1977 to 1981) to obtain further information on risks for different types of cancer and different ages at onset of disease. Paternal cigarette smoking emerged as a potential risk factor both for the generality of childhood cancer and for all ages at onset.


British Journal of Cancer (1997) 76(11), 1525–1531
© 1997 Cancer Research Campaign

### Childhood cancer and parental use of tobacco: deaths from 1971 to 1976

**T Sorahan[1], P Prior[2], RJ Lancashire[3], SP Faux[4], MA Hultén[5], IM Peck[1] and AM Stewart[3]**

[1]Institute of Occupational Health, University of Birmingham, Edgbaston, Birmingham B15 2TT; [2]Centre for Cancer Epidemiology, University of Manchester, Kinnaird Road, Manchester M20 4QL; [3]Department of Public Health and Epidemiology, University of Birmingham, Edgbaston, Birmingham B15 2TT; [4]MRC Toxicology Unit, Hodgkin Building, University of Leicester, PO Box 138, Lancaster Road, Leicester LE1 9HN; [5]LSF Research Unit, Regional Genetic Laboratory & Consultancy Services, Birmingham Heartlands Hospital NHS Trust, Yardley Green Rd, Birmingham B9 5PX, UK

**Summary** Parental smoking data have been reabstracted from the interview records of the Oxford Survey of Childhood Cancers (deaths from 1971 to 1976). Reported smoking habits for the parents of 2587 children who died with cancer were compared with similar information for the parents of 2587 healthy controls (matched pairs analysis). Maternal daily consumption of cigarettes and paternal use of pipes or cigars were unimportant, but there was a statistically significant positive trend between paternal daily consumption of cigarettes and the risk of childhood cancer ($P < 0.001$). This association could not be explained by maternal smoking, social class, parental ages at the birth of the survey child, sibship position or obstetric radiography. Relations between maternal consumption of cigarettes and birth weights suggested that (maternal) smoking data were equally reliable for case and control subjects. About 14% of all childhood cancers in this series could be attributable to paternal smoking. These data were combined with smoking data from two previously published reports from the Oxford Survey (deaths from 1953 to 1955, deaths from 1977 to 1981) to obtain further information on risks for different types of cancer and different ages at onset of disease. Paternal cigarette smoking emerged as a potential risk factor both for the generality of childhood cancer and for all ages at onset.

**Keywords:** childhood cancer; smoking; case-control study

The two largest studies of childhood cancer risks in relation to reported parental use of tobacco (combined series of 3190 cases) are based on data from the Oxford Survey of Childhood Cancers (OSCC) (Sorahan et al, 1995; 1997). Both studies found no significant association with maternal smoking habit and highly significant positive trends with paternal smoking habit. The more recent report also included summaries of 13 other published studies that provided information on childhood cancer risks in relation to paternal smoking (combined series of 2731 cases). A pooled estimate of risk (smokers vs non-smokers) indicated that results for fathers could not be easily dismissed as chance findings [relative risk (RR) 1.23, 95% confidence interval (CI) 1.14–1.33]. A wider confidence interval is obtained if the two OSCC reports are excluded (RR 1.20, 95% CI 1.07–1.35).

More recently, a US cohort study of 54 795 liveborn children found no association between childhood cancer risks before the age of 8 years and maternal use of cigarettes sometime during the pregnancy (RR 0.67, 95% CI 0.38–1.17, based on a total of 51 childhood cancers) (Klebanoff et al, 1996). Unfortunately, paternal smoking information was not collected as part of this study. One of the 13 studies referred to above was a research abstract (Ji et al, 1996), on which a full report confirming the results is now available (Ji et al, 1997).

Information on parental use of tobacco is only available for one further set of OSCC data (deaths from 1971 to 1976). These data have, therefore, been revisited to seek further information on the following hypothesis: paternal cigarette smoking is a risk factor for the overall grouping of all childhood cancers; paternal use of pipes or cigars and maternal cigarette smoking are unimportant.

*Received 22 April 1997*
*Revised 24 July 1997*
*Accepted 31 July 1997*

*Correspondence to:* T Sorahan

### MATERIALS AND METHODS

The OSCC, a national case–control study into the aetiology of childhood cancer, began in Oxford in 1955, but has been located at the University of Birmingham since 1975 (Stewart et al, 1958; Gilman et al, 1988). The survey has sought to interview the parents (usually the mother) of all children dying of solid cancers, leukaemia or allied malignant conditions before their sixteenth birthday in England, Wales and Scotland for the period 1953–84. A number of standard questionnaires, covering a wide range of social and medical topics, have been used during the course of this prolonged study. Data on parental smoking habits are not available for all years of the study.

There were 5111 childhood cancer deaths in England, Wales and Scotland for the period 1971–76. Interview data had been obtained from the parents of 2933 (57%) of these children. Parents of 819 case children had refused to participate with the survey, a further group of 428 case parents had moved abroad or to an unknown address, and the remaining 931 case parents had not replied to survey requests, their general practitioner had advised the survey not to approach them or arrangements to carry out interviews had fallen through. The overwhelming majority of the last group of case parents had not replied to survey requests; the response rate from case parents approached was thus at least 63% [2933/(5111–428)]. Some 25% of the interviewed case parents ($n$ = 642) had moved local authority area between the birth and death of the survey child. Some 97% of the interviews with case parents took place before the fourth anniversary of the death of the

**Table 1** Relative risks of childhood cancers for parental smoking habits, 1971–76 deaths, 2587 matched pairs

| Variable with levels | Cases | Controls | RR (95% CI) separate analysis of parental habits | RR (95% CI) simultaneous analysis of parental habits | RR (95% CI) additional adjustments[a] |
|---|---|---|---|---|---|
| *Cigarette smoking habit of mother* | | | | | |
| Non-smoker | 1367 | 1410 | 1.0 | 1.0 | 1.0 |
| 1–9 cpd[b] | 225 | 242 | 0.95 (0.78–1.16) | 0.94 (0.77–1.15) | 0.92 (0.75–1.13) |
| 10–19 cpd | 410 | 395 | 1.07 (0.92–1.26) | 1.01 (0.86–1.19) | 1.00 (0.85–1.19) |
| 20–29 cpd | 419 | 383 | 1.13 (0.96–1.32) | 1.03 (0.87–1.21) | 1.03 (0.87–1.22) |
| 30–39 cpd | 58 | 69 | 0.86 (0.60–1.24) | 0.79 (0.55–1.14) | 0.75 (0.52–1.09) |
| ≥ 40 cpd | 43 | 27 | 1.64* (1.01–2.67) | 1.42 (0.86–2.32) | 1.48 (0.89–2.44) |
| (*P*-value for trend)[c] | | | (0.151) | (0.910) | (0.909) |
| Smoker, amount n/k | 30 | 49 | 0.62* (0.38–0.99) | 0.57* (0.36–0.92) | 0.56* (0.35–0.91) |
| Smoking status n/k | 17 | 7 | 2.68* (1.05–6.86) | 2.24 (0.86–5.83) | 2.35 (0.89–6.20) |
| Ex-smoker[d] | 18 | 5 | 3.65* (1.36–9.85) | 3.75** (1.38–10.17) | 3.53* (1.28–9.75) |
| *Cigarette smoking habit of father* | | | | | |
| Non-smoker | 1008 | 1179 | 1.0 | 1.0 | 1.0 |
| 1–9 cpd | 118 | 139 | 0.99 (0.77–1.29) | 1.00 (0.77–1.30) | 1.02 (0.78–1.34) |
| 10–19 cpd | 326 | 289 | 1.33** (1.11–1.60) | 1.34** (1.11–1.61) | 1.37** (1.13–1.65) |
| 20–29 cpd | 579 | 533 | 1.30*** (1.12–1.51) | 1.29** (1.11–1.50) | 1.33*** (1.13–1.55) |
| 30–39 cpd | 157 | 133 | 1.43** (1.12–1.84) | 1.45** (1.12–1.87) | 1.42** (1.09–1.84) |
| ≥ 40 cpd | 144 | 105 | 1.62*** (1.24–2.11) | 1.61*** (1.22–2.11) | 1.63*** (1.23–2.15) |
| (*P*-value for trend)[c] | | | (< 0.001) | (< 0.001) | (< 0.001) |
| Smoker, amount n/k | 105 | 103 | 1.19 (0.89–1.60) | 1.18 (0.88–1.59) | 1.25 (0.92–1.69) |
| Ex-smoker[d] | 22 | 15 | 1.79 (0.92–3.48) | 1.87 (0.95–3.69) | 2.12* (1.06–4.23) |
| Smoking status n/k | 128 | 91 | 1.73*** (1.29–2.34) | 1.65** (1.21–2.23) | 1.99*** (1.44–2.76) |

*$P < 0.05$, **$P < 0.01$, ***$P < 0.001$. [a]Parental smoking habits analysed simultaneously with social class (five levels: I, II, III, IV, V), age of father at birth of survey child (six levels: < 20, 20–24, 25–29, 30–34, 35–39, ≥ 40), age of mother at birth of survey child (six levels: < 20, 20–24, 25–29, 30–34, 35–39, ≥ 40), sibship position (five levels: 1, 2, 3, 4, ≥ 5), and obstetric radiography (yes/no); [b]cpd = cigarettes per day; [c]trend over first six levels only, i.e. ignoring n/k categories; [d]stopped smoking at least 2 years before birth of survey child child (median interval, 21 months). The median interval between the birth of the case child and the parental interview was 8 years and 5 months.

For each case child with interview data, a 'control list' of six children, matched for sex and date of birth, was selected from the birth register of the local authority area in which the case child died. Control parents were contacted in turn until one control family agreed to be interviewed. Interview data were obtained for 2628 control children (1371 first choices, 472 second choices and 785 later choices). (Control interviews were not obtained for 305 case children with interview data; these cases do not feature in the analysis.) Only 52% of first choices may seem a low percentage but the birth registers from which the controls were selected had been compiled, on average, some 8 or 9 years before the interviews were arranged. The case and control parents within each pair were interviewed by the same person, usually a physician or nurse from the local health authority.

For the purpose of this report, the interview folders of all matched pairs were reviewed and information on parental use of tobacco was reabstracted and amalgamated with existing study computer files. A preinterview form (postal questionnaire) had been sent to those parents (cases and controls) who agreed to participate in the survey which asked, 'Do you smoke? If YES, please say about how much each day'. The main interview questionnaire requested information on 'smoking' (from all participating parents) in terms of 'daily quantity'; the question was also directed at current rather than past smoking habits. Information was abstracted in terms of daily consumption of cigarettes, use of pipe and use of cigars. When the daily consumption of cigarettes was reported with upper and lower values, the upper value was selected. Given that all the smoking questions were directed at current habits, there was no requirement for ex-smokers to identify themselves. A small number did so, and for these analyses, ex-smokers were defined as parents who stopped smoking at least 2 years before the survey child was born (23 mothers and 37 fathers). Other ex-smokers were included with the smokers (i.e. smokers in the 2 year period before birth of the survey child). A response limited to ounces of tobacco was assumed to relate to a pipe smoker. A total of 79 mothers and 208 fathers were reported to be smokers but no information on daily consumption was supplied. The smoking questions were left unanswered for a further group of 24 mothers and 219 fathers; most of these fathers were not living with their children.

Birth weight data were reabstracted for each child, birth weights obtained from obstetric clinic records were allowed to take precedence over the weights given by mothers.

After excluding 41 matched pairs in which the case child was adopted, case and control data relating to tobacco consumption (2587 matched pairs) were compared (with and without adjustment for other variables) by means of (multiple) conditional logistic regression using the EGRET program. Smoking habits of mothers and fathers were analysed separately, simultaneously, and simultaneously with additional adjustment for other variables. The purpose of the simultaneous analyses was to allow for the effects of other variables, so that the independent effects of each smoking habit could be examined. The odds ratio was used to obtain estimates of relative risk (RR). Risks are shown relative to a baseline risk of unity for the non-smokers.

© Cancer Research Campaign 1997

**Table 2** Relative risks of childhood cancers by type of tumour associated with smoking habits of parents, 1971–76 deaths

| | | Smoking habit of mother[a] | | Smoking habit of father[a] | |
|---|---|---|---|---|---|
| **Type of tumour** | **Matched pairs** | **RR[b]** | **(95% CI)** | **RR[b]** | **(95% CI)** |
| Acute lymphatic leukaemia | 573 | 0.98 | (0.89–1.07) | 1.07 | (0.99–1.16) |
| Myeloid leukaemia | 190 | 1.00 | (0.83–1.20) | 1.27** | (1.10–1.47) |
| Monocytic leukaemia | 25 | 0.66 | (0.36–1.19) | 0.84 | (0.56–1.26) |
| Other and unspecified leukaemia | 47 | 0.91 | (0.67–1.24) | 0.99 | (0.75–1.30) |
| Lymphoma | 165 | 1.05 | (0.89–1.23) | 1.07 | (0.92–1.23) |
| Wilms' tumour | 87 | 0.83 | (0.63–1.09) | 1.12 | (0.91–1.38) |
| CNS cancers | 410 | 1.07 | (0.95–1.19) | 1.02 | (0.93–1.11) |
| Neuroblastoma | 193 | 0.97 | (0.82–1.14) | 1.13 | (0.99–1.29) |
| Bone cancers | 91 | 1.08 | (0.87–1.35) | 1.09 | (0.90–1.32) |
| Other solid cancers | 206 | 0.99 | (0.84–1.16) | 1.13 | (0.99–1.29) |
| Benign tumours | 141 | 0.99 | (0.82–1.20) | 1.23* | (1.05–1.44) |
| All diagnoses | 2128 | 0.99 | (0.95–1.04) | 1.09*** | (1.05–1.14) |

*$P < 0.05$, **$P < 0.01$, ***$P < 0.001$. [a]Only first six levels of smoking habit (see Table 1) are considered; the n/k categories are ignored. Levels are coded 1–6 and the variable is treated as a continuous variable. Maternal and paternal habits are analysed simultaneously. [b]These relative risks (unadjusted for other variables) refer to a change of one level for smoking habit; a relative risk which is significantly different from unity indicates a statistically significant trend of risk with smoking habit.

**Table 3** Relative risks of childhood cancer by cigarette smoking habits of one or both parents

| **Cigarette smoking habits** | **Cases** | **Controls** | **RR** | **(95% CI)** | **RR additional adjustments[a]** | **(95% CI)** |
|---|---|---|---|---|---|---|
| Neither parent | 704 | 804 | 1.0 | | 1.0 | |
| Mother only | 323 | 390 | 0.95 | (0.80–1.14) | 0.94 | (0.78–1.12) |
| Father only | 630 | 573 | 1.27** | (1.09–1.48) | 1.29** | (1.10–1.51) |
| Both parents | 792 | 727 | 1.26** | (1.09–1.46) | 1.27** | (1.09–1.48) |
| n/k | 138 | 93 | 1.79*** | (1.33–2.41) | 2.12*** | (1.54–2.92) |

* $P < 0.05$, ** $P < 0.01$, *** $P < 0.001$. [a]See footnote a, Table 1.

### RESULTS

Relative risks for all types of childhood cancers combined are shown by parental cigarette smoking habits in Table 1. Smoking habits of mothers and fathers are first analysed separately (two analyses), then simultaneously (one further analysis), and then simultaneously with additional adjustment for parental ages at the birth of the survey child, social class (based on occupation of father), sibship position, and obstetric radiography (one further analysis). None of the point estimates of relative risk shown for five categories of daily maternal smoking habit in the final column of Table 1 was statistically significant, although there was a significantly reduced risk for 'smoker, amount not known' (RR 0.56, 95% CI 0.35–0.91) and a significantly elevated risk for ex-smokers (RR 3.53, 95% CI 1.28–9.75). There were significantly elevated risks for four out of the five categories of daily paternal smoking habit (10–19, 20–29, 30–39, ≥ 40 cpd). For fathers, highest point estimates of relative risk were shown for 'smoking status not known' (RR 1.99, 95% CI 1.44–2.76) and ex-smokers (RR 2.12, 95% CI 1.06–4.23). There was no significant trend between amount of maternal smoking (six levels, nil to ≥ 40 cpd) and childhood cancer risk ($P = 0.91$), whereas the corresponding trend for paternal smoking was highly significant ($P < 0.001$). Neither the use of a pipe by fathers (160 cases, 152 controls, RR 1.06, 95% CI 0.84–1.33) nor the use of cigars by fathers (67 cases, 68 controls, RR 0.98, 95% CI 0.69–1.40) was associated with elevated risks of childhood cancer (these unadjusted relative risks were obtained from separate analyses and are not shown in Table 1).

The analysis summarized in the final column of Table 1 was repeated for those 1945 matched pairs in which the case parents (and by definition, the control parents) had not moved local authority area between the birth and death of the survey child. A significant positive trend ($P < 0.001$) was obtained (point estimates of relative risk for paternal cigarette smoking were as follows: 1–9 cpd, 0.95; 10–19 cpd, 1.32; 20–29 cpd, 1.25, 30–39 cpd, 1.47; ≥ 40 cpd, 1.55).

Relative risks associated with paternal and maternal daily smoking habit are shown for eleven diagnostic groups in Table 2. To enable a summary to be given in a single table, relative risks for a change of one smoking level are provided. A total of 21 out of the 22 confidence intervals shown for site-specific relative risks include the corresponding point estimate of relative risk for all types of childhood cancers combined. Formal tests indicated that there was no significant heterogeneity in site-specific relative risks for either mothers or fathers.

The role of interactions between maternal and paternal habits is examined in Table 3, which shows relative risks for use of cigarettes by neither parent, mother only, father only and both parents, with and without the adjustments described above. Statistically significant risks are shown for father only and for both parents; the point

© Cancer Research Campaign 1997

**Table 4** Mean birthweight of case and control children by parental cigarette smoking habits

| Daily cigarette consumption | Mean birthweight in ounces[a] (and number of children) | | | | | | | |
|---|---|---|---|---|---|---|---|---|
| | Mother | | | | Father | | | |
| | Case | | Control | | Case | | Control | |
| Non-smoker | 121.2 | (1357) | 120.4 | (1408) | 120.2 | (1003) | 119.1 | (1177) |
| < 10 cpd | 118.9 | (222) | 118.3 | (242) | 117.3 | (116) | 119.0 | (139) |
| 10–19 cpd | 116.5 | (408) | 115.1 | (394) | 118.4 | (323) | 118.2 | (289) |
| 20–29 cpd | 114.6 | (419) | 114.5 | (381) | 118.3 | (574) | 117.6 | (533) |
| 30–39 cpd | 112.9 | (58) | 113.1 | (69) | 114.7 | (157) | 117.1 | (132) |
| > 40 cpd | 117.6 | (42) | 111.7 | (27) | 122.8 | (143) | 117.2 | (104) |
| Smoker, amount n/k | 123.7 | (29) | 121.7 | (49) | 119.3 | (104) | 116.2 | (103) |
| Ex-smokers | 119.3 | (18) | 128.6 | (5) | 121.6 | (22) | 117.5 | (15) |
| Smoking status n/k | 118.5 | (11) | 122.6 | (7) | 113.9 | (122) | 116.8 | (90) |
| Total | 118.9 | (2564) | 118.6 | (2582) | 118.9 | (2564) | 118.6 | (2582) |

[a]Original units were pounds and ounces.

**Table 5** Relative risks of childhood cancers, by type of tumour, associated with cigarette smoking by parents: 5777 matched pairs (1953–55 deaths, 1971–76 deaths, 1977–81 deaths)

| Type of tumour | Matched pairs | Mothers | | | | | | Fathers | | | | | |
|---|---|---|---|---|---|---|---|---|---|---|---|---|---|
| | | Non-Smoker | | Smoker | | | | Non-Smoker | | Smoker | | | |
| | | Case | Control | Case | Control | RR[a] | (95% CI) | Case | Control | Case | Control | RR[a] | (95% CI) |
| Leukaemia | 2364 | 1257 | 1285 | 1055 | 1032 | 1.02 | (0.90 to 1.16) | 779 | 863 | 1475 | 1418 | 1.20* | (1.05 to 1.37) |
| Lymphoma | 503 | 258 | 273 | 228 | 220 | 0.96 | (0.73 to 1.27) | 162 | 208 | 314 | 269 | 1.67*** | (1.23 to 2.26) |
| Wilms' tumour | 278 | 162 | 140 | 114 | 133 | 0.67* | (0.46 to 0.99) | 91 | 93 | 180 | 174 | 1.27 | (0.85 to 1.92) |
| CNS cancers | 1071 | 559 | 599 | 484 | 459 | 1.01 | (0.84 to 1.23) | 356 | 416 | 660 | 619 | 1.30* | (1.06 to 1.59) |
| Neuroblastoma | 472 | 233 | 241 | 224 | 221 | 0.95 | (0.71 to 1.26) | 149 | 191 | 302 | 264 | 2.02*** | (1.45 to 2.82) |
| Bone cancers | 232 | 114 | 132 | 113 | 98 | 1.31 | (0.87 to 2.00) | 74 | 89 | 152 | 136 | 1.24 | (0.80 to 1.93) |
| Other solid cancers | 584 | 303 | 324 | 270 | 247 | 1.17 | (0.91 to 1.52) | 190 | 226 | 362 | 341 | 1.30 | (0.98 to 1.74) |
| Benign tumours | 273 | 138 | 155 | 128 | 114 | 1.31 | (0.87 to 1.99) | 102 | 127 | 156 | 138 | 1.31 | (0.88 to 1.94) |
| All diagnoses | 5777 | 3024 | 3149 | 2616 | 2524 | 1.02 | (0.94 to 1.10) | 1903 | 2213 | 3601 | 3359 | 1.29*** | (1.19 to 1.41) |

*$P < 0.05$, **$P < 0.01$, ***$P < 0.001$. [a]Parental smoking habits analysed simultaneously with social class (five levels: I, II, III, IV–V, not known), age of father at birth of survey child (five levels: < 24, 25–29, 30–34, 35–39, ≥ 40), age of mother at birth of survey child (six levels: < 20, 20–24, 25–29, 30–34, 35–39, ≥ 40), sibship position (five levels: 1, 2, 3, 4, ≥ 5), and obstetric radiography (yes/no). These relative risks refer to smoker/non-smoker comparisons; the analyses included two other smoking categories for which relative risks are not shown in the Table (ex-smokers: 67 case and 42 control mothers, 72 case and 54 control fathers; smoking status not known: 70 case and 62 control mothers, 201 case and 151 control fathers.

estimate of relative risk for both parents (1.27) was similar to that for father only (1.29). Consequently, Table 3 provides no evidence of an important interaction between maternal and paternal habits.

Information on the reliability of the smoking data was sought from a separate examination of the data relative to birth weights. Maternal smoking is known, from other sources, to produce low birth weights, (US Department of Health and Human Services, 1980). Mean birth weights, by level of parental cigarette consumption are shown in Table 4. Among both case and control groups there were negative trends ($P < 0.001$) for birth weight with maternal daily consumption of cigarettes; similar trends were not found for paternal smoking habits. The effects of three variables (case/control status, maternal use of cigarettes, paternal use of cigarettes) on birth weight were examined in an analysis of variance. Only maternal consumption of cigarettes made a statistically significant contribution ($P < 0.001$) to explaining the variance in the birth weight variable.

Data on deaths for 1971–76 were combined with smoking data from two previously published OSCC reports (1549 matched pairs relating to deaths from 1953 to 1955 (Sorahan et al, 1997) and 1641 matched pairs relating to deaths from 1977 to 1981 (Sorahan et al, 1995)) to obtain further information on risks for different types of cancer and different ages at onset of disease. Relative risks associated with parental daily smoking habits, and obtained from the combined series, are shown for eight diagnostic groups in Table 5. To enable a summary to be given in a single table, relative risks are provided for smokers compared with non-smokers (these risks are not directly comparable, therefore, with the risks shown in Table 2). A total of 14 out of the 16 confidence intervals shown for site-specific relative risks include the corresponding point estimate of relative risk for all types of childhood cancers combined. Formal tests indicated that there was no significant heterogeneity in site-specific relative risks for either mothers or fathers.

 *© Cancer Research Campaign 1997*

**Table 6** Relative risks of childhood cancers for paternal cigarette smoking by age at onset of disease: 5777 matched pairs (1953–55 deaths, 1971–76 deaths and 1977–81 deaths)

| Age-group (y) | RES neoplasms[a] | | | Solid cancers | | | All diagnoses | | |
|---|---|---|---|---|---|---|---|---|---|
| | Matched pairs | RR[b] | (95% CI) | Matched pairs | RR[b] | (95% CI) | Matched pairs | RR[b] | (95% CI) |
| 0–1 | 512 | 1.22 | (0.92–1.49) | 835 | 1.26 | (1.01–1.58) | 1347 | 1.22 | (1.03–1.45) |
| 2–3 | 741 | 1.28 | (1.01–1.63) | 558 | 1.35 | (1.02–1.79) | 1299 | 1.32 | (1.10–1.58) |
| 4–5 | 516 | 1.06 | (0.80–1.40) | 468 | 1.79 | (1.31–2.43) | 984 | 1.35 | (1.10–1.65) |
| 6–7 | 356 | 1.39 | (0.96–2.02) | 317 | 1.25 | (0.85–1.84) | 673 | 1.25 | (0.96–1.62) |
| 8–9 | 300 | 1.64 | (1.11–2.43) | 247 | 1.33 | (0.84–1.84) | 547 | 1.49 | (1.12–1.98) |
| 10–11 | 170 | 1.33 | (0.82–2.16) | 216 | 1.12 | (0.70–1.80) | 386 | 1.20 | (0.88–1.64) |
| 12–13 | 182 | 1.35 | (0.81–2.25) | 181 | 1.01 | (0.63–1.64) | 363 | 1.15 | (0.82–1.61) |
| 14–15 | 90 | 1.75 | (0.82–3.71) | 88 | 2.19 | (1.04–4.64) | 178 | 1.98 | (1.21–3.25) |
| Linear[c] | | $P = 0.13$ | | | $P = 0.25$ | | | $P = 0.89$ | |
| Quadratic[d] | | $P = 0.23$ | | | $P = 0.21$ | | | $P = 0.88$ | |

[a]RES = reticulo-endothelial system. [b]Paternal smoking habits analysed simultaneously with social class (five levels: I, II, III, IV–V, not known), age of father at birth of survey child (five levels: < 24, 25–29, 30–34, 35–39, ≥ 40), age of mother at birth of survey child (six levels: < 20, 20–24, 25–29, 30–34, 35–39, ≥ 40), sibship position (five levels: 1, 2, 3, 4, ≥ 5), and obstetric radiography (yes/no). These relative risks refer to smoker/non-smoker comparisons; the analyses included two other smoking categories for which relative risks are not shown in the Table (ex-smokers: 72 case and 54 control fathers; smoking status not known: 201 case and 151 control fathers. [c]*P*-value for linear component of the interaction between age at onset of disease and paternal smoking. [d]*P*-value for quadratic component of the interaction between age at onset of disease and paternal smoking.

Relative risks, also obtained from the combined series, for paternal cigarette smoking (smokers vs non-smokers) in relation to three diagnostic groupings (all neoplasms of the reticuloendothelial system, all solid cancers and all cancers) and eight categories of age at onset of disease (as judged by the parents) are shown in Table 6. There is no obvious pattern in the displayed relative risks and formal analyses of linear and quadratic components in the pattern of relative risks failed to identify any significant departures from homogeneity in any of the three sets of data. A similar analysis of relative risks by age at death (using the same eight age categories) also produced a null result. A separate analysis (not shown in Tables) failed to show any significant linear or quadratic components in the interaction between paternal smoking and paternal age at the birth of the child.

### DISCUSSION

The study provides further supportive evidence of an association between the smoking of cigarettes by fathers and cancer in their offspring; the smoking of cigarettes by mothers can, with some confidence, be excluded as an important risk factor for the generality of childhood cancers.

If the paternal smoking association is causal in nature, this might be due either to preconception effects or to the effects of passive smoking on young infants or both. A passive smoking effect seems unlikely because of the weight of evidence against maternal smoking being a risk factor for childhood cancers; it might be imagined that, in general, the infant has more contact with passive smoke from the mother than from the father. A preconception effect is not biologically implausible and evidence for potential mechanisms have been reviewed (Wyrobek, 1993; Wyrobek and Adler, 1996; Woodall and Ames, 1997).

From measurements of in vitro cultured blood lymphocytes, it is well known that smokers have a significantly increased frequency of genetic abnormalities (van Diemen et al, 1995; Zaire et al, 1996). Smoking would be expected to induce chromosome and DNA mutations in the germ line. There are, however, important differences between oogenesis and spermatogenesis because stem cell proliferation in the female takes place during fetal life only, whereas spermatogenesis is ongoing throughout adult life. Spermatogenesis, a specialized process taking 72–74 days to complete, involves premeiotic stem cell proliferation and differentiation from type A to type B spermatogonia, followed by the two meiotic divisions and finalized by the maturation of testicular spermatids to spermatozoa (Adler 1996). Substantial chromatin modification takes place during spermiogenesis and the repair activity of cells is considerably reduced during the final weeks of this process. To our knowledge, no direct investigations of smoking effects have been performed on the genetic material of premeiotic cells, the spermatogonia, meiotic spermatocytes or post-meiotic cells. However, some information is available on oxidative damage to sperm DNA in smokers.

Cigarette smoke contains a high concentration of oxidants that deplete tissues and seminal fluid of those antioxidants that would normally neutralize the damaging species (Schectman et al, 1989). If unchecked, oxidants can cause considerable damage to lipids, proteins and DNA and these reactive mutagens have been shown to be involved in a variety of physiological processes, including cancer (Ames et al, 1993). Evidence that smoking increases oxidative damage to sperm DNA is available from measurements of steady state levels of $oxo^8dG$ in sperm DNA, an index of oxidative damage (Fraga et al, 1996). These studies showed that levels of $oxo^8dG$ were some 50% higher in the DNA isolated from the sperm of smokers than basal levels of $oxo^8dG$ in sperm DNA from non-smokers. Oxidative lesions are found in all sperm; in non-smokers they are caused by endogenous processes. In addition, a reduction in antioxidant levels in seminal fluid was also reported for the group of smokers. The findings of Fraga et al (1996) are consistent with the hypothesis that adequate antioxidant protection is essential to minimize the risk of mutations and maintain the genetic integrity of sperm cells (Fraga et al, 1991), and that smoking comprimises the oxidant–antioxidant balance. It is possible, of course, that other mechanisms may account for any paternal sperm mutation.

© *Cancer Research Campaign 1997*

There is no reason to believe that any risk presented by paternal smoking before conception would only affect one type of childhood cancer. On the basis of the combined OSCC reports, our results suggest that the risk factor may be operating across the spectrum of childhood cancers. Risks may be more pronounced for lymphomas and neuroblastomas, although much of the variation in the ranking of site-specific risks from study to study may represent no more than chance fluctuations. It does not follow, of course, that each and every subtype of childhood cancer is necessarily affected by paternal smoking. There is also no reason to believe that any risks presented by paternal smoking before conception would be confined to cancer risks. Information on paternal smoking and congenital anomalies are available from one study of 17 152 births from the three largest obstetric units in west Jerusalem in the period November 1974 to December 1976 (Seidman et al, 1990). A monotonic non-significant positive trend ($P = 0.15$, calculated by present authors) was shown for incidence of congenital anomalies (major or minor) and paternal smoking habit (non smoker: 73.5 per 1000 births; ≥ 30 cpd: 76.7 per 1000 births; < 30 cpd: 85.2 per 1000 births). These rates are based on 9838, 6140 and 1174 births respectively.

The paternal results are most unlikely to be due to chance because in each of the three relevant OSCC studies, trends with smoking habit have been highly significant ($P < 0.001$). Confounding also presents an unlikely sole explanation. The potential confounders which have been considered in the new data (social class, age of father, 'family mobility' etc.) had little effect on the paternal smoking findings and the use of alcohol can be excluded on the basis of previous work (Sorahan et al, 1995; Ji et al, 1997). If an unknown variable was confounding the paternal smoking effect, it would need, by definition, to be associated with higher risks than paternal smoking, both for point estimates of relative risk and for attributable risk. The confounder would, therefore, need to be responsible for some 15% (or more) of all childhood cancers; an unusual occupational exposure would not, therefore, provide a likely candidate.

One key issue in evaluating the importance of these findings is the reliability of OSCC data. For the data relating to mothers' smoking habits there was one test of their reliability, namely the relation with birth weight. For the fathers' smoking habits there was no similar test, although the very different findings for the use of pipes, cigars and cigarettes suggests that there was no general misrepresentation of fathers' smoking habits. A comparison with data on the prevalence of cigarette smoking in Great Britain, obtained from Table 6.3 of the 1990 General Household Survey (OPCS, 1992), indicated that only case fathers had an elevated prevalence. The percentages of survey parents who were cigarette smokers were compared with national (expected) percentages, adjusting for sex, age at interview (16–19, 20–24, 25–34, 35–49, 50–59, ≥ 60) and year of interview (2-year intervals). Observed and expected percentages of smokers in case fathers were 58.1% and 52.2% respectively. Corresponding percentages for other parents were as follows: control fathers, 52.2% and 52.0%; case mothers: 46.1% and 46.4%; control mothers: 45.2% and 46.1%. These comparisons suggest that the paternal smoking effect is not an artefact caused by the control fathers having an unusually low prevalence of smokers. However, the possibility of differential reporting of cigarette smoking habits between case and control fathers remains.

Other issues need to be considered. The new data are limited by the modest response rate, and the effects of having to ignore the non-responders are not known. The method of selecting controls means that 'mobile' families tend to be under-represented in the control series although analyses restricted to 'non-mobile' families suggested that this feature of control selection was not an important issue for this analysis The case series in this study comprised childhood cancer deaths rather than all incident cases, and some improvement in survival rates past the age of 16 years did take place in the later survey years (Sorahan and Roberts, 1993). The inclusion of childhood cancer survivors could have led to materially different results if paternal smoking only increased mortality rates in children diagnosed with cancer. It would be difficult to maintain such a hypothesis given that the paternal smoking findings were reasonably consistent across calendar periods. Before these analyses being carried out, it had been predicted that a paternal smoking effect would be more pronounced for younger ages at presentation of childhood cancer, and that bias would offer an unlikely explanation for such a finding. No evidence of such an effect was found. It could be argued that the paternal findings in the new series merely reflect changes in paternal smoking brought on by the death of a child. However, a change in alcohol consumption would seem even more likely, and as mentioned above, there is no evidence for a paternal alcohol effect. Caution is still required, however, in interpreting these findings because it is not possible to exclude all potential biases from the findings.

More information on the subject is required. The paternal smoking data available to many case–control studies of childhood cancer have not yet been fully analysed and reported. Even more useful would be the results of new, large case–control studies; also valuable would be analyses of cancer in the offspring of subjects whose smoking habits were collected in contexts other than case–control studies. If the relative risks provided by this third set of OSCC data are accurate, then approximately 14% of all childhood cancers might be attributable to paternal smoking; the corresponding percentage for the three OSCC reports combined is 13%.

### ACKNOWLEDGEMENTS

We thank Jaswant Bal and Suvineetha Wanasundara for the abstraction of birth weight data. We thank Dr Estelle Gilman for assistance with the 'mobility' analyses and Linda Hamilton for assistance with the calculation of pooled risk estimates. TS receives generous financial support from The Colt Foundation. PP received support from the Cancer Research Campaign. MAH receives support from the Department of Health for a CHARR project, registered by the Health and Safety Executive as project RSU no. 54.056

### REFERENCES

Adler ID (1996) Comparison of the duration of spermatogenesis between male rodents and humans. *Mutat Res* **352**: 169–172

Ames BN, Shigenaga MK and Hagan TM (1993) Oxidants, and antioxidants, and the degenerative diseases of aging. *Proc Natl Acad Sci USA* **90**: 7915–7922

Van Diemen PCM, Maasdam D, Vermeulen S, Darroudi F and Natarajan AT (1995) Influence of smoking habits on the frequencies of structural and numerical chromosomal aberrations in human peripheral blood lymphocytes using the fluorescence in situ hybidization (FISH) technique. *Mutagenesis* **10**: 487–495

Fraga CG, Motchnik PA, Shigenaga MK, Helbock HJ, Jacob RA and Ames BN (1991) Ascorbic acid protects against endogenous oxidative damage in human sperm. *Proc Natl Acad Sci USA* **88**: 11003–11006

Fraga CG, Motchnik PA, Wyrobek AJ, Rempel DM and Ames BN (1996) Smoking and low antioxidant levels increase oxidative damage to sperm DNA. *Mutat Res* **351**: 199–2032

*© Cancer Research Campaign 1997*

Gilman EA, Kneale GW, Knox EG and Stewart AM (1988) Pregnancy X-rays and childhood cancers: effects of exposure age and radiation dose. *J Soc Radiol Prot* **8**: 9–18

Ji BT, Shu XO, Linet MS, Zheng W, Ying DM and Jin F (1996) Paternal pre-conception cigarette smoking and the risk of childhood cancer. *Am J Epidemiol* **143**: S86 (suppl.)

Ji BT, Shu XO, Linet MS, Zheng W, Wacholder S, Gao YT, Ying DM and Jin F (1997) Paternal pre-conception cigarette smoking and the risk of childhood cancer in the offspring of nonsmoking mothers. *J Natl Cancer Inst* **89**: 238–244

Klebanoff MA, Clemens JD and Read JS (1996) Maternal smoking during pregnancy and childhood cancer. *Am J Epidemiol* **144**: 1028–1033

Office of Population Censuses and Surveys (1992) General Household Survey 1990. HMSO: London.

Schectman G, Byrd JC and Grucho H (1989) The influence of smoking on vitamin C status in adults. *Am J Pub Health* **79**: 158–162

Seidman DS, Ever-Hadani P, Gale R (1990) Effect of maternal smoking and age on congenital anomalies. *Obstet Gynecol* **76**: 1046–1050

Sorahan T and Roberts PJ (1993) Childhood cancer and paternal exposure to ionizing radiation: preliminary findings from the Oxford Survey of Childhood Cancers. *Am J Ind Med* **23**: 343–354

Sorahan T, Lancashire R, Prior P, Peck I and Stewart A (1995) Childhood cancer and parental use of alcohol and tobacco. *Ann Epidemiol* **5**: 354–359

Sorahan T, Lancashire R, Hulten MA, Peck I and Stewart AM (1997) Childhood cancer and parental use of tobacco: deaths from 1953 to 1955. *Br J Cancer* **75**: 134–138

Stewart AM, Webb J and Hewitt D (1958) A survey of childhood malignancies. *Br Med J* **i**: 1495–1508

Woodall AA and Ames BN (1997) Nutritional prevention of DNA damage to sperm and consequent risk reduction in birth defects and cancer in offspring. In *Preventive Nutrition: The Comprehensive Guide for Health Professionals*. Bendich A and Deckelbaum RJ (eds), Humana Press: Totowa, NJ, USA

Wyrobek AJ (1993) Methods and concepts in detecting abnormal reproductive outcomes of paternal origin. *Reprod Toxicol* **7** (suppl.) **1**: 3–16

Wyrobek AJ and Adler ID (1996) Detection of aneuploidy in human and rodent sperm using FISH and applications of sperm assays of genetic-damage in heritable risk-evaluation. *Mutat Res* **352**: 173–179

Zaire R, Grittin CS, Simpson PJ, Papworth DG, Savage JRK, Armstrong S and Hultén MA (1996) Analysis of lymphocytes from uranium mineworkers in Namibia for chromosomal damage using Fluorescence in situ Hybridization (FISH). *Mutat Res* **371**: 109–113

© Cancer Research Campaign 1997